# 

**DOI:** 10.1192/bjb.2022.48

**Published:** 2023-06

**Authors:** Femi Oyebode

**Affiliations:** Honorary Professor of Psychiatry and a consultant psychiatrist in the Institute of Clinical Sciences, University of Birmingham, UK. Email: femi_oyebode@msn.com

**Figure:**
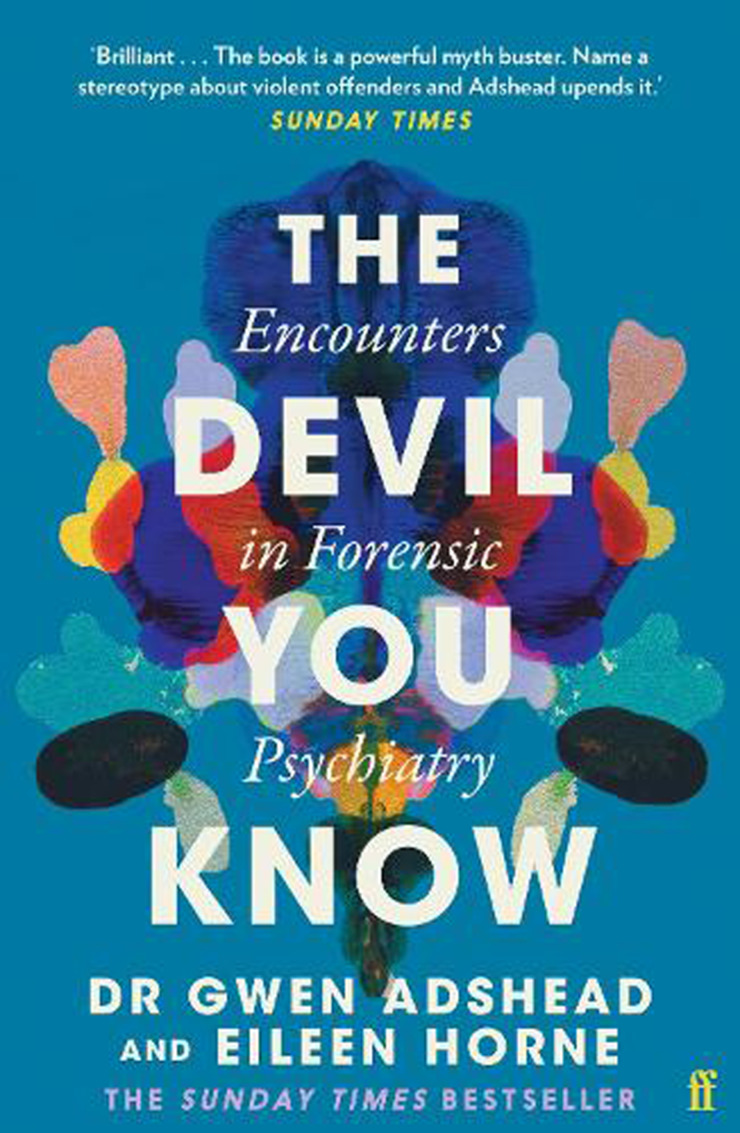

Sartre in his novel *Nausea* writes about privileged moments in the encounter between people. These moments have the potentiality of something emerging from a particular set of circumstances and are conceived as having a rare and precious quality. In Gwen Adshead's wonderful account of working as a psychotherapist within forensic psychiatry, several privileged moments are described, many of which are pivotal in transforming what might initially be experienced as impasses into truly meaningful interactions. The book chapters are named after particular individuals, Tony, Gabriel, Kezia and so on, and the method is to situate the clinical encounters in their actual settings, be it at Broadmoor Hospital, a private clinic room, a prison or elsewhere. This takes the clinical case history to a literary dimension, facilitating our appreciation of the setting of the encounter, the appearance of the person and their demeanour but also of Adshead's own feelings and responses to the encounter. The fragility of the person and often the vulnerability of Adshead herself give the account a freshness and honesty not often encountered in clinical accounts of mental illness and its impact on life. This is even more impressive given that many, but not all, of the people under consideration and scrutiny have committed serious offences, including murder.

There are several examples of attentive listening and attunement to the momentous statement that is simply expressed. Marcus for example says ‘All I wanted was to be beautiful’ and Adshead is able to demonstrate the associative thinking that is the cornerstone of clinical reasoning, and of psychotherapy in particular, drawing allusions to one of the core features of ‘incel’ beliefs. Another clinical skill that is well illustrated is the power of clinical observation, shown for example in Adshead's description of Charlotte: ‘looking at her I was reminded of myself at fourteen or so, sitting in just that posture, hunched on my bed with arms folded and head down, full of ennui and attitude when asked to do something I didn't want to do’. One is awed by the use of similes by the patients: Charlotte in response to the question ‘what does anger feel like to you?’ replied ‘it feels like dragon's breath when it comes’. And there is the use of metaphor by Adshead herself: ‘I like to think of the mind as a coral reef, mysterious and complex, ever metabolising, teeming with things of beauty and danger'.

If ever there was doubt that there is an aesthetic of clinical encounters, then reading Horne and Adshead's book should quickly relieve that doubt. There are several references to literature, including Shakespeare and poetry, as means of deepening our understanding of troubling situations. This is a book for all clinicians and also for the public. It shows psychiatry off in the best possible light, but it is not anodyne.

